# Post-mortem diagnosis of imported malaria in France: a case report

**DOI:** 10.1186/s12936-021-03806-y

**Published:** 2021-06-14

**Authors:** Jacques Sevestre, Caroline Bernardi, Morgane Gillet, Pascal Delaunay, Youta Fanjat, Giorgio Toni, Pierre Marty, Véronique Alunni, Christelle Pomares

**Affiliations:** 1 Service de Parasitologie-Mycologie, Hôpital L’Archet, Centre Hospitalier Universitaire de Nice, UCA, 151 route de Saint Antoine de Giestière, 06000 Nice, France; 2 Laboratoire de Médecine Légale Et Anthropologie Médico-Légale, Hôpital Cimiez, Centre Hospitalier Universitaire de Nice, Nice, France; 3 Laboratoire Central D’Anatomie Pathologique, Hôpital Pasteur, Centre Hospitalier Universitaire de Nice, Nice, France; 4 Equipe 6 Virulence Microbienne Et Signalisation Inflammatoire, C3M, INSERM 1065, Nice, France

**Keywords:** Malaria, *Plasmodium*, Cerebral malaria, Malaria diagnosis, Malaria prevention, Travel medicine, Case report

## Abstract

**Background:**

Malaria is a potentially lethal parasitic disease due to infection by *Plasmodium* parasites, transmitted by *Anopheles* mosquito vectors. Various preventative measures may be recommended for travellers who visit endemic areas. The diagnosis is generally evoked in the context of a febrile patient returning from an endemic zone. Nevertheless, symptoms and clinical signs may be difficult to interpret, and fatal cases may only be diagnosed retrospectively with laboratory techniques, specific pathological features and patient history. The present work reports a case of fatal cerebral malaria diagnosed post-mortem, along with the techniques that allowed identification of the causative agent.

**Case presentation:**

A 29 year-old male was found dead in his rental home during a vacation in Southern France. In the absence of explainable cause, an autopsy was performed, which did not retrieve major lesions. In the context of frequent business-related travels in tropical Africa, several samples were adressed for parasitological examination. Microscopy techniques, along with immunochromatographic and molecular biology assays, led to post-mortem diagnosis of fatal cerebral malaria. It was discovered in retrospect that the patient had not used preventative measures against malaria when travelling in endemic zones, and had not been provided with proper travel medicine counseling prior to his travel.

**Conclusion:**

A vast proportion of imported malaria cases reported in France concerns patients who did not use preventive measures, such as bed nets, repellents or chemoprophylaxis. Given the wide availability of prevention tools in developed countries, and the important number of declared imported malaria cases, there is no doubt traveller awareness still needs to be raised. Moreover, healthcare professionals should always question travel history in febrile patients. The authors advocate for recurrent information campaigns for travellers, and physician training for a better prevention and diagnosis of malaria cases.

## Background

Malaria is a potentially lethal parasitic disease due to *Plasmodium* sp*.* infection, transmitted by *Anopheles* mosquito vectors. Even though a lot of progress has been made in the battle against malaria, it still accounts for a considerable number of deaths worldwide, estimated around 440,000 in 2018 [1] Among all these casualties, an overwhelming proportion occurs in the African continent, where several *Plasmodium* species are endemic, with *Plasmodium falciparum* being the most frequent one. This species is also the main culprit for lethal malaria cases [1]. The recent progress in the fight against malaria are the result of a broad distribution of long-lasting insecticidal mosquito nets, as well as the availability of efficient diagnostic tests and treatments [2, 3]. Most people who live in endemic areas develop a partially protective immunity (i.e. premunition) over time. However, travellers who are naïve for the parasite may develop a fatal infection [1]. Thus, preventative measures are generally enforced for travellers in malaria-endemic countries. Travel medicine specialists offer different options according to the risk level: mosquito nets, repellents and chemoprophylaxis (CP), which reduce the risk of infection [4]. Nevertheless, none of these measures ensures 100% protection. Once infection has starded, clinical signs and symptoms are nonspecific: a high temperature, headhaches, vomiting, diarrrhoea, chills, muscle pains and fatigue [1]. They may be difficult to recognize, and physicians do not always think of the possibility of a *Plasmodium* infection if they are not aware of the patient’s travel history. The present work report a case of post-mortem diagnosed malaria case in a man who regularly travelled in endemic zones for professional purposes.

## Case presentation

**Fig. 1:**
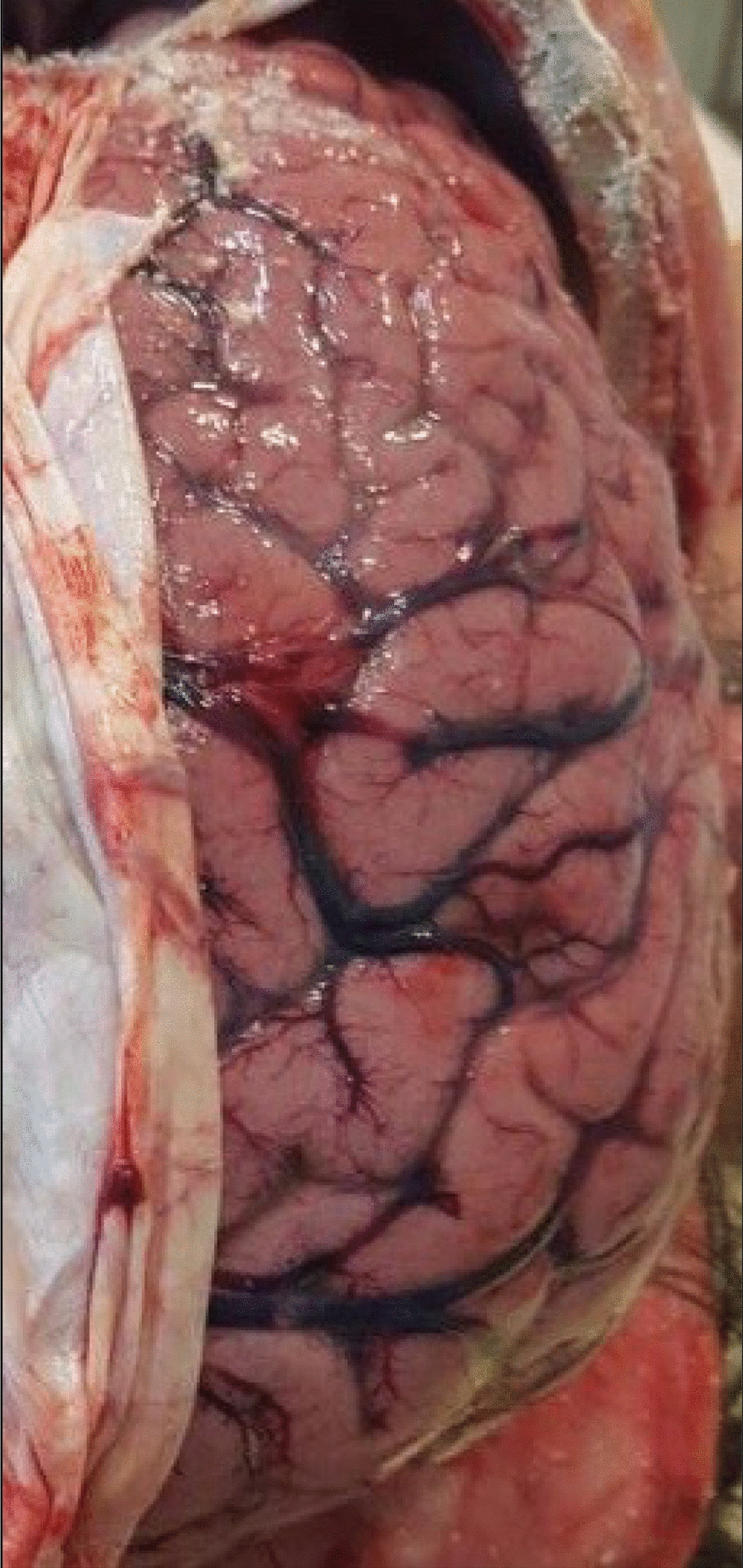
Autopsic aspect of the right parietal lobe of the brain, showing oedema, with widening of the gyri and narrowing of the sulci, and congestion

This case study concerns a 29 year-old Caucasian male of Dutch nationality who was found dead on a rental holiday home in July 2018 in southern France. Forensic autopsy findings were notable for oedema and congestion of the brain (Fig. 1), and splenomegaly. In the absence of major macroscopic lesion, no conclusions were drawn from forensic examination.

Upon questioning, family members explained that the man had no known medical condition. He worked a salesman for a food industry company, and had visited many African countries for professional purposes, travelling to Benin, Gambia, Guinea, the Ivory Coast, Liberia, Mali, Niger, Tchad, Togo and Senegal in the previous year. His last trip had occured a month before his death, in Guinea, where he had spent five days. Moreover, his family explained that he reported feeling sick for the past four months, with recurrent fever, headache, nausea and diarrhoea. They also mentioned that over this four-month period during which he experienced symptoms, he had visited several general practitioners in his hometown in the Netherlands. He presented with intermittent, febrile gastrointestinal symptoms and headaches associated with great fatigue. He had been prescribed an oral course of antibiotics, which did not improve his symptoms, and common blood chemistry analysis performed three months before his death only retrieved a slight elevation of Alanine Amino-Transferase (ALAT) measured at 56 U/l (above a reference range of 0 to 41). All other parameters were within normal range. No diagnostic tests for *Plasmodium* infection had been prescribed.

**Fig. 2:**
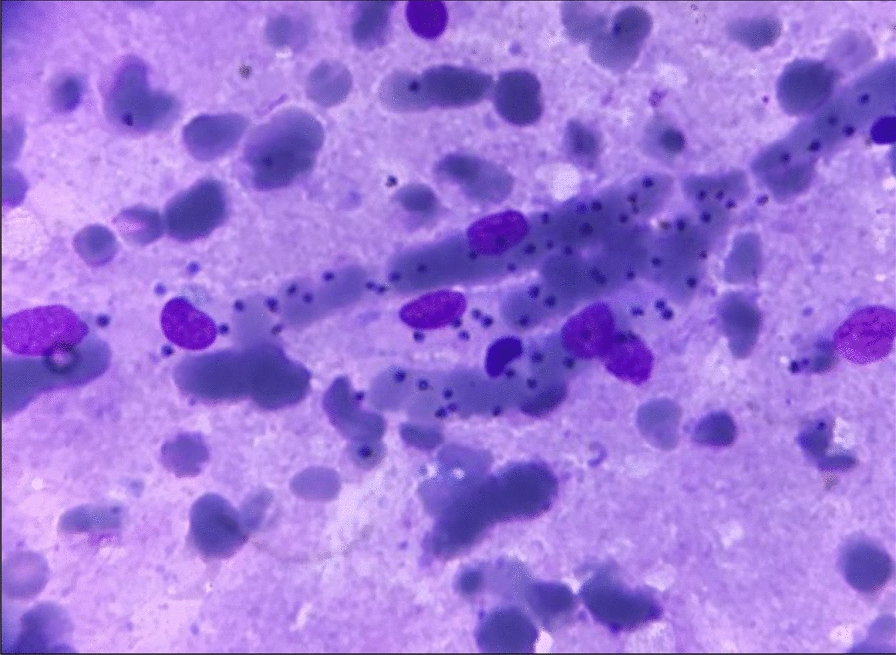
Microscopic aspect of a dry smear obtained from brain tissue apposition. Numerous trophozoïtes can be observed in the path of a small vessel (May-Grünwald-Giemsa staining, x1000)

**Fig. 3:**
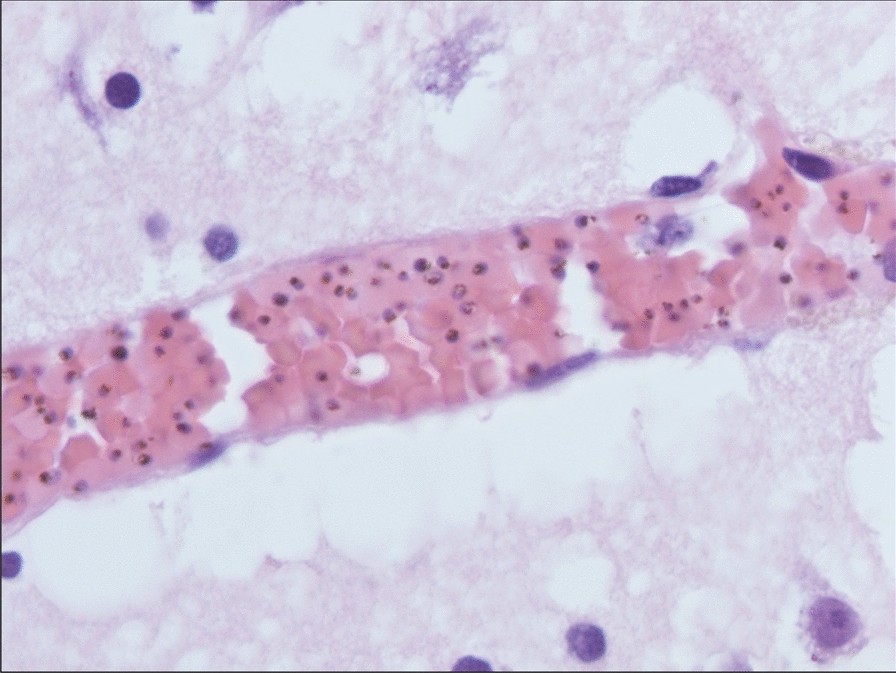
Brain parenchyma section showing intercellular oedema and venular congestion, with numerous parasitized erythrocytes. (Hematoxylin-Eosin-Saffron staining, x200)

Post-mortem, samples from various body tissues were subjected to pathological and parasitological examination. Parasitological examination was performed on peripheral blood, brain tissue and spleen samples. Microscopic examination of peripheral blood smears and dry smears from the apposition of brain and spleen tissue stained with May-Grünwald-Giemsa (MGG) retrieved numerous trophozoïtes of *P. falciparum* (Fig. 2). In peripheral blood, parasitaemia was estimated at 5%. An immunochromatographic rapid diagnostic test (RDT) (PALUTOP + 4, BioSynex, Illkirch, France) performed on peripheral blood showed positivity for *Plasmodium* Lactate Dehydrogenase and Histidine Rich Protein-2 antigens, which was also consistent with *P. falciparum* infection. Pathological examination of brain tissue stained with the Haematoxylin–Eosin-Saffron (HES) method was consistent with previous findings, revealing numerous parasitized erythrocytes in the cerebral veins lumen (Fig. 3). Real-time polymerase chain reaction (PCR) analysis was secondarily performed by the French National Reference Centre in Marseille, France, and confirmed the presence of *P. falciparum* DNA in peripheral blood samples. Altogether, these results led to a post-mortem diagnosis of fatal cerebral imported malaria.

In retrospect, it was found that this man had not been properly informed about risks regarding business travels in malaria-endemic countries. Indeed, he had not seen any travel medicine professional prior to his several travels in Africa, nor was he prescribed CP or repellents. However, he had received vaccination against yellow fever.

## Discussion and conclusion

The present work reports a case of fatal cerebral imported malaria in a man who had probably been infected for months prior to this dramatic outcome. This report summarizes several features for the diagnosis of malaria, and how post-mortem investigations may lead to retrospective diagnosis of a fatal complicated form, with cerebral involvement.

Malaria is generally separated in two distinct clinical presentations: uncomplicated and severe. Uncomplicated malaria clinical signs are non-specific such as “flu-like" symptoms, fever, headaches, and also nauseas and diarrhoea [1]. Severe malaria follows uncomplicated malaria and may involve different vital functions, the most frequent being cerebral malaria, acute respiratory distress syndrome, acute kidney failure and acidosis [1]. Even when treated, cerebral malaria fatality rates may reach 10–20% in adults, highlighting the severity of this clinical form [1, 5].

For this patient, the diagnosis of cerebral malaria was done post-mortem. In this context, examination of the brain generally reveals unspecific autopsic signs, such as oedema, congestion and petechias [6, 7]. Techniques commonly used in clinical microbiology, such as peripheral smears and RDTs are of great aid, given the rapidity of the results and the high specificity for the diagnosis of malaria [8]. Histological findings may also orient towards a diagnosis of cerebral malaria. Histopathological features of cerebral malaria generally include vascular congestion, parasitized erythrocyte sequestration, ring trophozoïtes and the presence of malarial pigment in the small capillary vessels [6, 8, 9]. In this patient, gross autopsy findings were not conclusive, whereas rapid smear techniques (e.g. dry smears and thin blood smears) and RDTs quickly prompted the diagnosis of fatal cerebral malaria, which was secondarily confirmed by histopathological findings and PCR.

In France, 2,840 cases of imported malaria have been recorded in 2018 [10]. Lethal cases only occurred in a small proportion (11 cases = 0.38%) and were all due to *P. falciparum* infection [10]. Ten out of the 11 patients did not use proper preventative measures against malaria, whereas one patient had taken an inappropriate CP [10]. Dutch national surveillance data showed that 1,941 malaria cases were recorded between 2008 and 2015, including seven fatal outcomes, and underlined an increase in reported cases in this period of time [11].

This case tragically highlights several points that should remain etched in the mind of every medical professional. Before any travel in a malaria endemic area, traveller awareness still needs to be raised by health professionals and authorities, as almost all the imported malaria cases reported in France either did not observe chemoprophylaxis or used it inappropriately [4]. Patient information and education may considerably lower the risk of being infected during a stay in endemic zone [4, 12]. Once travellers return from endemic areas, physicians should always consider the eventuality of malarial infection in a febrile patient, even after several months and whatever the symptoms. They should never hesitate to test patients for malaria infection, given the relative simpleness and rapidity of diagnostic techniques [1].

In this man's history, several opportunities to avoid the fatal outcome were missed. First, he was not provided with counseling by a travel medicine specialist, and no CP was initiated. Then, none of the physicians consulted by the patient after his travels were able to make a diagnosis of malaria. General practitioners should always question a possible history of travel in patients presenting with fever or unspecific signs. Indeed, in malaria-free areas, the key to securing diagnosis in febrile subjects is to unveil a history of travel in endemic regions [1].

In developed countries, travellers have an easy access to travel medicine practitioners. Furthermore, online resources for malaria prevention are generally available for physicians and travellers in developed countries [13–16]. An important proportion (if not all) of the imported malaria casualties could be avoided with adapted prevention.

## Data Availability

Not applicable.
